# Epigenetic Regulation in Knee Osteoarthritis

**DOI:** 10.3389/fgene.2022.942982

**Published:** 2022-07-08

**Authors:** Zhengyu Cai, Teng Long, Yaochao Zhao, Ruixin Lin, You Wang

**Affiliations:** Department of Bone and Joint Surgery, Renji Hospital, School of Medicine, Shanghai Jiao Tong University, Shanghai, China

**Keywords:** epignetics, knee osteoarthritis, DNA methylation, histone modification, noncoding RNA

## Abstract

Osteoarthritis (OA) is a complicated disease with both hereditary and environmental causes. Despite an increase in reports of possible OA risk loci, it has become clear that genetics is not the sole cause of osteoarthritis. Epigenetics, which can be triggered by environmental influences and result in transcriptional alterations, may have a role in OA pathogenesis. The majority of recent research on the epigenetics of OA has been focused on DNA methylation, histone modification, and non-coding RNAs. However, this study will explore epigenetic regulation in OA at the present stage. How genetics, environmental variables, and epigenetics interact will be researched, shedding light for future studies. Their possible interaction and control processes open up new avenues for the development of innovative osteoarthritis treatment and diagnostic techniques.

## Introduction

**FIGURE 1:**
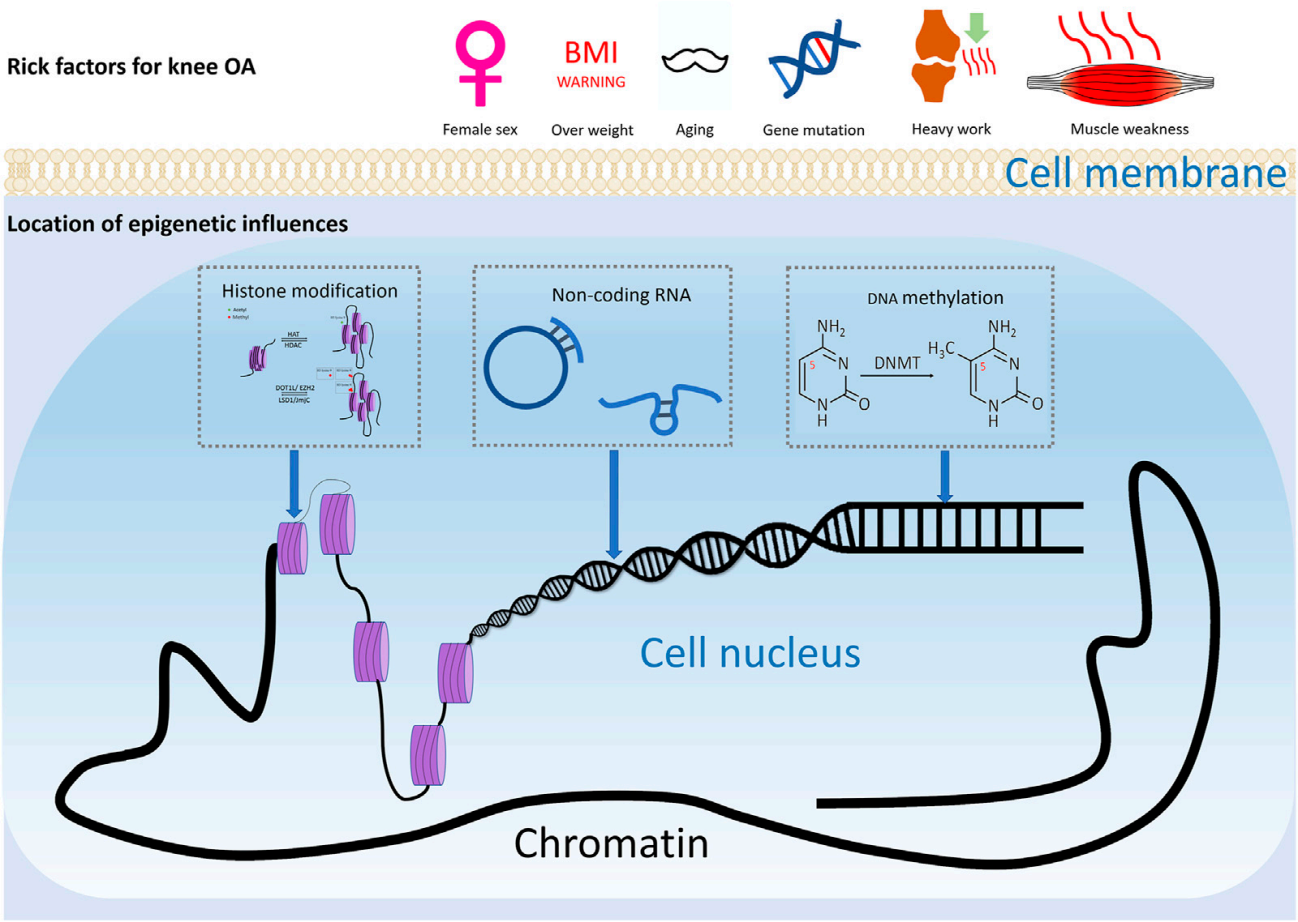
Risk factors of knee OA. Risk factors for knee osteoarthritis includes ageing, gender, injury, and joint overloading, etc. Epigenetics may play a considerable role in how these environmental factors lead to altered gene expression and ultimately pathophysiologic manifestations such as cartilage damage and subchondral osteosclerosis.

Osteoarthritis (OA) is a widespread joint disease that affects around 15% of the world’s population (Glyn-Jones et al., 2015). OA was once thought to be a degenerative illness arose from chronic wear-and-tear and mechanical stress. However, the emerging paradigm now sees it as a complex process involving interactions between a wide range of internal and environmental factors (Figure 1). The genetic component of the disease is complex. Current evidence supports the theory of a polygenic inheritance, as published studies have reported a variety of OA-related risk loci (Styrkarsdottir et al., 2018; Zengini et al., 2018; Styrkarsdottir et al., 2019; Tachmazidou et al., 2019). However, studies have shown that more than 80% of the disease-related variants are located in non-coding regions of the genome (Maurano et al., 2012). As a result, it has been proposed that changes in gene expression, rather than changes in the genetic code sequence, are more likely to influence OA development. Indeed, epigenetics, a crucial method of gene expression regulation, has been implicated in the start and progression of OA in recent research (Ramos and Meulenbelt, 2017).

Epigenetics is an essential gene-environment interaction process. Epigenetic modification, unlike genomic modifications, is more versatile and reversible. Regulations of epigenetics vary by cell type and gene. Epigenetic phenomena including DNA methylation, genomic imprinting, maternal effects, post-translational modifications of histones, RNA (controlled by non-coding regulatory RNAs), and epigenetic chromatin remodeling (three-dimensional structure of chromatin) have been well studied. (Simon and Jeffries, 2017). With the help of modern testing techniques, epigenetic studies of knee osteoarthritis are now possible to reveal how the external environment affects changes in somatic cells and tissues. Many studies have been conducted on the epigenetics of osteoarthritis, although they have primarily focused on DNA methylation, histone modification, and miRNA (Ramos and Meulenbelt, 2017). This section will review the epigenetic regulation and reciprocity based on previous findings, trying to seek the connection, investigate how genetics and epigenetics interact, and steer future research toward identifying biologically significant alterations and gaining a better understanding of the mechanisms involved.

## DNA Methylation

**FIGURE 2:**
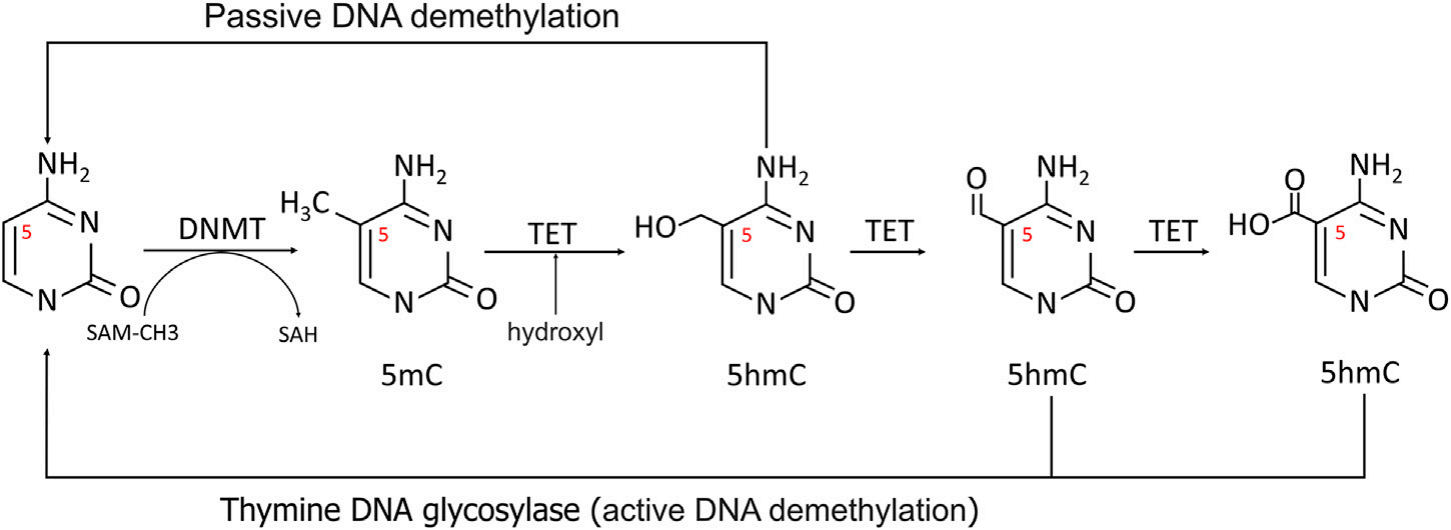
Methylation and demethylation of DNA. In methylation process, the 5′carbon of cytosine was methylated to 5-MC with S-adenosine methionine (SAM) as the methyl donor. During active demethylation, the methyl group of 5 mC can be modified by the TETs-mediated addition of hydroxyl groups to generate 5-hydroxymethylcytosine (5hmC), which can be subsequently processed in demethylation process.

The most fully studied methylation is 5-methylcytosine (5 mC) (Miranda-Duarte, 2018)**.** CpG dinucleotides are distributed unevenly throughout the human genome, with the bulk of CpG islands found in the promoter and exon regions of genes 300 to 3000bp in length. As a result, the methylation level of CpGs can be dynamically controlled by DNMTs and TETs to regulate transcription (Figure 2) (Rice et al., 2018).

Recent research into global and gene-specific methylation has proved a clear link between methylation and the development of OA. Helmtrud I. et al. were the first to study individual methylation loci. They investigated the amount of methylation of proximal promoter regions of aggrecanases1 (*ADAMTS 4*) and matrix metalloproteinase (*MMPs*) 2 (gelatinase A), *MMP3* (stromelysin 1), *MMP9* (gelatinase B), and *MMP13* (collagenase 3) (Roach et al., 2005). Despite the fact that the demethylation and sensitivity to demethylation of these four enzymes differed significantly, the degree of demethylation of at least one CpG site in each enzyme in OA was statistically higher than in the control group.

Additional DNA methylation locations encompassing numerous OA-related chemicals or pathways have been discovered in subsequent cartilage tissue studies, and these genes can be classified into four main categories. The first category addresses the extracellular matrix’s homeostasis, which includes *COL2A1, COL9A1, COL10A1, ACAN, MMP2, MMP3, MMP9, MMP13, ADAMTS-4* (Pöschl et al., 2005; Roach et al., 2005; Dickhut et al., 2009; Imagawa et al., 2014). Inflammation-related molecules, such as *IL1b, IL8, IL32, TGF-β2, IL1RN, and WNT11,* fall under the second category (Hashimoto et al., 2009; Moazedi-Fuerst et al., 2014; Takahashi et al., 2015). *SOX4, SOX9, RUNX2, and SOD2* are members of the third group, which are involved in cartilage maintenance (Ezura et al., 2009; Kim et al., 2013). Growth factors such as *BMP7, SOST, and GDF5* fall within the fourth category (Loeser et al., 2009; Reynard et al., 2011; Papathanasiou et al., 2015). DNA methylation is known to vary greatly between tissues and stages of illness. Such studies only identify methylation sites that may be related to disease due to sampling issues. Still, these findings add to the current understanding of OA epigenetics.

### CpG Methylation Patterns Correlated With OA-Associated SNPs

**FIGURE 3:**
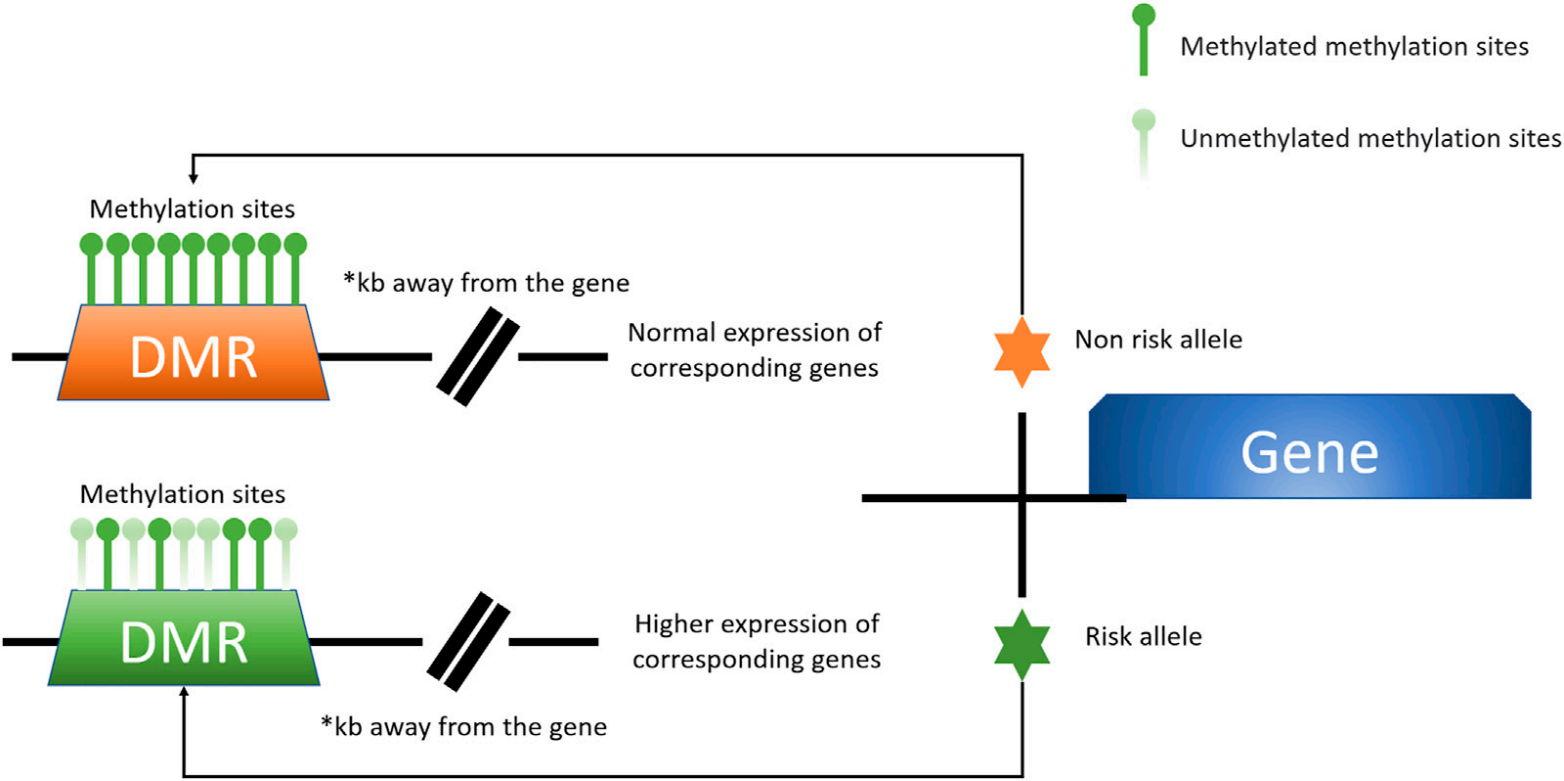
A model for the presence of altered levels of DMR methylation due to specific risk alleles is shown here. Certain OA-associated SNP mutations may lead to alterations in the degree of DMR methylation at a distance from the associated causative gene, which affects the expression of the associated gene. This unique mode of action is better studied and defined in the locus of chromosome 6p21.1 labelled rs10948172, with the associated gene RUNX2 (Zhang G. et al., 2020).

Direct SNP correlations or gene-environment interactions may be mediated by these methylation quantitative trait loci (meQTLs). Eilis H. et al. investigated DNA methylation at 850,000 locations across the genome using a MethylationEPIC BeadChip (850K chip) in samples from the Understanding Society UK Household Longitudinal Study (UKHLS) (n = 1,111) (Hannon et al., 2018). They discovered 548 significant DNA methylation quantitative trait loci correlations between 2,907,234 genetic variations and 93,268 DNA methylation sites, resulting in 548 significant DNA methylation quantitative trait loci. They offered two key concepts: 1) mQTL-associated SNP mutations were more common within 500 kb of the DNA methylation site (designated as cis), and the cis SNP variation had a much more considerable influence on DNA methylation; 2) proximal methylation sites shared mQTL-associated SNP mutations. Differential methylation region refers to the methylation region regulated by such SNPs (DMR). These results explained the association between SNP changes and surrounding DMR in the proximal regions of several genes correlated with OA risk in osteoarthritis (*RUNX2*, *PLEC*, *ALDH1A2*, *GDF5*, *MGP*, *COLgalt2*, and *COL11A2*) (Figure 3) (Reynard et al., 2014; Shepherd et al., 2018; Rice et al., 2019a; Shepherd et al., 2019). Further mQTL research revealed 24 CpGs linked to genes including *COLgalt2*, *COL11A2*, *RAPH1, FGFR3* and *WWP2*. Importantly, *WWP2* encloses miRNA 140, which have been shown to be critical in cartilage maintenance, as miRNA 140 have severe cartilage defects (Duan et al., 2020). These findings demonstrate that DNA methylation can influence gene and non-coding RNA expression, as well as gene transcription and translation (Table 1).

**TABLE 1:** Genes with a differentially methylated region potentially related to single nucleotide polymorphism

OA Associated SNP	CpG loci	Potential gene	
rs6976	cg15147215	cg18099,408	*GNL3* Rushton et al. (2015)
	cg18591801		
rs10948172	cg20913747	cg13979708	*RUNX2* Rushton et al. (2015); Rice et al. (2019a)
	cg18551225	cg19254793	
rs3204689	cg12031962	-	*ALDH1A2* Rushton et al. (2015)
rs143383	cg14752227	-	*GDF5* Rushton et al. (2015)
rs10471753	cg25008444	-	*PIK3R1* Rice et al. (2019a)
rs11780978	cg02331830	cg19405177	*PLEC*
	cg04255391	cg20784950	*GRINA* Rice et al. (2019a)
	cg14598846	cg01870834	
	cg23299254	cg07427475	
	cg10299941	-	
rs4764133	cg20917083	-	*MGP* Rice et al. (2019a)
rs6516886	cg20220242	cg00065302	*RWDD2B* Rice et al. (2019a)
	cg24751378	cg05468028	
	cg16140273	cg18001427	
rs11583641	cg18131582	-	*COLGALT2* Rice et al. (2019b)
rs62182810	cg10114877	-	*NBEAL1* Rice et al. (2019b)
rs11732213	cg25007799	cg20987369	*FGFR3* Rice et al. (2019b)
rs9277552	cg13921245	cg02197634	*COL11A2* Rice et al. (2019b)
	cg02375585	cg25491704	
rs60890741	cg18170545	-	*ASAP1* Rice et al. (2019b)
rs317630	cg22375663	-	*CPSF1* Rice et al. (2019b)
rs35206230	cg10253484	cg20040747	*SEMA7A* Rice et al. (2019b)
rs6499244	cg26736200	-	*NFAT5* Rice et al. (2019b)
	cg26661922		*WWP2* Rice et al. (2019b)
rs2953013	cg16779580	-	*RAB11FIP4*
rs62063281	cg16520312	cg17117718	*LRRc37A*
	cg18228076	cg10826688	*CRHR1*
	cg01934064	cg15295732	*MAPT*
	cg15633388	cg11117266	*KANSL1* Rice et al. (2019b)
	cg23616531	-	-
rs11583641	cg18131582	-	*COLGALT2* Kehayova et al. (2021)
rs6516886	cg20220242	-	*RWDD2B* Parker et al. (2021)
rs75621460	Not mentioned in paper	-	*TGFβ1* Rice et al. (2021)

### Risk Factors and DNA Methylation in OA

Animal models have provided light on the relationship between recognized OA risk factors like injury, aging and altered methylation patterns in the formation of OA. Destabilization of the medial meniscus, for example, can lead to OA development and altered 5mc and 5hmc patterns in 12-week-old male mice (Singh et al., 2019). Furthermore, tamoxifen-induced articular chondrocyte specific deletion of *DNMT3b* (*AGCCRE* ^*ERT2*^ *; DNMT3b* ^*fl/fl*^), the *de novo* methyltransferase, can result in OA-like phenotypes in knee joints; whereas *DNMT3b* overexpression in articular chondrocytes can delay OA development after meniscus ligament injury (Shen et al., 2017).

Other OA risk factors, such as obesity, appear to be mediated by DNA methylation. Obesity has long been assumed to cause OA due to increased mechanical loading and wear and tear. Recent evidence shows that even in non-weight-bearing joints, such as hands, obesity still seems to be a significant risk factor. Leptin, a cytokine-like peptide hormone secreted by white adipose tissue, has long been stipulated to connect obesity with OA. Because obese patients generally have a high level of leptin in their bodies, their blood level is regulated significantly by body fat content. This is most likely owing to the obese patient’s altered methylation pattern in the leptin (*lep*) promoter region. According to a recent study, patients after bariatric surgery have lower serum leptin levels and a different methylation profile in the lep and lepr gene promoter regions. These findings back up previous studies in rat models, which indicated that a high-energy diet could alter *lep* methylation patterns (Milagro et al., 2009). While inhibiting leptin signaling in ob^−/−^ and DB^−/−^ mice models resulted in obesity, it did not affect the occurrence of knee OA (Griffin et al., 2014). The suppression of *lep* methylation by 5′-Aza-2-deoxycytidine (AZA) increased MMP13 activity in patient chondrocytes *in vitro*. *MMP-13* was elevated after leptin’s epigenetic reactivation, and small interfering RNA against lep inhibited it directly (Iliopoulos et al., 2007). Overall, existing evidence suggests that leptin methylation could link obesity and OA development.

### The Role of DNA Methyltransferase in OA

Members of the *DNMTs* family include *DNMT1, DNMT2, DNMT3a, DNMT3b,* and *DNMT3l*. The main function of *DNMT2* is to catalyze the methylation of RNA, while *DNMT3l* does not have catalytic activity. CpG methylation is determined by three DNA methyltransferases: *DNMT1, DNMT3A, and DNMT3B. DNMT1* is responsible for maintaining methylation during DNA replication and damage repair. *DNMT3A* and *DNMT3B* play major role in *de novo* methylation (Gao L. et al., 2020). A previous matched case-control study found a connection between DNMT polymorphisms and primary knee OA. Under a co-dominant and dominant paradigm, the CT haplotype of DNMT1 polymorphisms was related to a lower risk of OA. In contrast, the CC genotype of rs2424913 of DNMT3b was associated with an increased risk (Miranda-Duarte et al., 2020).

Differential expression of *DNMT1* in human chondrocytes collected from different areas in articular chondrocytes can be triggered by IL-1, a pro-inflammatory cytokine. Notably, deep and superficial zone chondrocytes, instead of transition zone chondrocytes, enhanced DNMT1 protein expression and activity in response to IL-1 (Akhtar and Haqqi, 2013). The demethylation of *DMNT1* by AZA can greatly increase the expression of pro-inflammatory or matrix carbolic proteins such as *COX2, MMP9, and MMP13*. Only *DNMT3b* was expressed in the mature normal cartilage of the knee joint and TMJ, but not *DNMT3a* or *DNMT1*. Additionally, and the occurrence of OA in both joints would lead to the decrease of *DNMT3b* expression (Shen et al., 2017). Overexpression of DNMT3b also appears to slow the onset of osteoarthritis caused by trauma or chemicals (Shen et al., 2017; Zhou et al., 2019).

## Histone Modification

The most in-depth study on OA’s overall accessible chromatin landscape change was performed by comparing cartilage from both the outer region of the lateral tibial plateau and the inner region of the medial tibial plateau from the same patient. The accessible chromatin landscapes of injured and undamaged tissues display strikingly distinct chromatin signatures, notwithstanding patient-to-patient differences (Liu et al., 2018). Further analysis revealed that enhancers account for the majority of differentially accessible peaks, including enhancers from *BMPR1b, WNT5a, and FGFR2*, all of which are known to play a role in OA-related cellular activity. Histone modification is shown to regulate some essential chondrocyte regulator genes, such as SOX9, as well as functioning in conjunction with epigenetic regulators to affect downstream genes (Kim et al., 2013). SOX11 and WNT5b, two OA-related genes, had differing accessible peaks in their promoter regions.

### Histone Acetylation in OA

**FIGURE 4:**
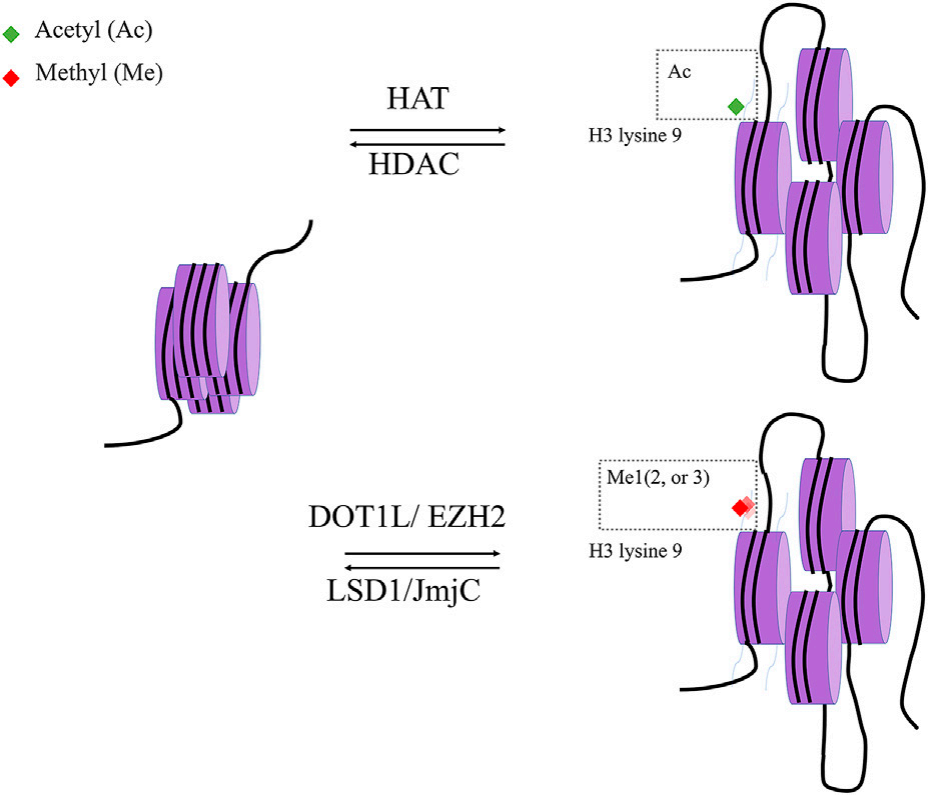
histone modification. Histone acetylase (HAT) and Histone deacetylase (HDAC) are two classes of enzymes responsible for the structural modification of chromosomes and regulation of gene expression. In general, acetylation of histones facilitates the dissociation of DNA from histone octamers and the relaxation of nucleosome structure, thus allowing various transcription factors and co-transcription factors to bind specifically to DNA binding sites and activate gene transcription. In contrast, deacetylation of histones exerts the opposite effect with Histone acetylase.

Histone acetylation is a dynamic process governed by two distinct enzyme families: HATs and HDACs. HDACs can modify nonhistone proteins to affect a variety of cellular processes in addition to their effects on chromatin structure (Figure 4).

Currently, most research on HDACs’ functions in OA pathogenesis shows their interactions with non-histone proteins, which change target gene transcription. HDAC1 and HDAC2 were detected in large amounts in human chondrocytes and synovial cells from OA patients. They may alter the expression of cartilage-specific genes such as *COL9A1, COL11A1, COMP, AGGRECAN, and Dermatopontin*, likely *via* interaction with *SNAIL* protein (Hong et al., 2009). *HDAC4*, which is not indicated in normal knee cartilage, was highly expressed in OA cartilage in other research employing knee cartilage specimens from OA patients and normal donors. modulate *JNK* and *ERK1/2* activation to mediate *IL1b*-induced matrix catabolic protein synthesis (Wang et al., 2018). The *HDAC9-PIASy-RNF4* axis promotes chondrocyte hypertrophy by regulating the sumo and ubiquitination of Nkx3.2/Bapx1, which is degraded by the proteasome, according to studies on *HDAC9* in osteoarthritis (Choi et al., 2016).

A few studies on the regulation of mesenchymal stem cell destiny discloses that the epigenetic role of *HDAC* may also be involved in the development of OA. In rate bone marrow stromal cells, *HDAC8* can inhibit osteogenesis via two pathways: it can inhibit histone H3 lysine 9 acetylation, which reduces the osteogentic protein *RUNX2*, Osterix, Osteopotin and ALP. It can also associate with *RUNX2* to repress its transcriptional activity (Lee et al., 2019). Human MSCs increased global histone acetylation following *in situ* stiffening in a hydrogel environment by reducing *HDAC* activity. Histone acetylation and Lamin A/C, the mechanosensor signaling protein, were considerably low in subchondral bone isolated from OA patients, while *HDAC* activity was significantly higher (Lee et al., 2019).

Sirtuins (*SIRTs*) are histone and protein deacetylases dependent on nicotinamide dinucleotide (NAD+). SIRTs and SIRT-dependent epigenetic control have been widely reported to play vital roles in DNA repair, inflammation, and aging-related illnesses (Lee et al., 2019). Chondrocyte hypertrophy and proteoglycan production were reduced in *SIRT6*-deficient mice from the mesenchyme (Ailixiding et al., 2015). Sirtuin 1 (*SIRT1*), like *SOX9*, is essential for maintaining cartilage homeostasis (Tsuda et al., 2003). Boosted *SIRT1* expression increased the expression of cartilage ECM genes such as *COL2A1, COL9A1, AGGRECAN* and *COMP*. SIRT1 has been found to connect with *SOX9* and *p300*, *GN5*, both of which are involved in nucleosome acetylation in the *COL2* promoter region. *SIRT3* is mostly found in mitochondria, and its deacetylation activity regulates mitochondrial function, regeneration, and kinetics (Ansari et al., 2017). These activities are hypothesized to maintain REDOX equilibrium in cell metabolism, preventing oxidative stress (Bause and Haigis, 2013).

Studies concentrating on deacetylase/acetylases still have significant limitations because *HATs/HDACs* can modify transcription factors as well as signaling molecules, thus affecting the expression of other distant genes (Zhao et al., 2008). *HDAC* inhibitors, for example, can stimulate the acetylation of other proteins in several physiological pathways in addition to increasing histone acetylation. The non-targeted protein deacetylase inhibitors Zolinza (Vorinostat) and Istodax (Romidepsin) have been approved for the treatment of cutaneous T-cell lymphoma. Histone modification medicines for osteoarthritis, on the other hand, have not yet been developed.

### Histone Methylation

Typically, histone methylation is more stable than histone acetylation. Histone methylation is performed by histone methylation transferase (HMT) on lysine and arginine residues. Common locations of methylation include Lys 4, 9, 27, 36, 79 and Arg 2, 17, 26 on H3, Lys 20 and Arg 3 on H4, and Lys 4, 9, 27, 36, 79 and Arg 2, 17, 26 on H3 and H4. Studies have shown that arginine methylation on histone is a relatively dynamic marker. Arginine methylation is associated with gene activation, whereas arginine methylation loss in H3 and H4 is linked to gene silence (Greer and Shi, 2012). In contrast, lysine methylation appears to be a reasonably persistent marker for gene expression regulation.

Similar to histone acetylation, changes in histone methylation are frequently related to altered gene expression and signaling pathways in chondrocytes. For instance, H3k79 methylation was reduced in OA and RA patients (He et al., 2017), while H3K9 methylation was decreased in the temporomandibular joints of elderly mice (Ukita et al., 2020). DOT1L, an enzyme involved in histone methylation of Lys79 of H3 (H3K79), was a cartilage homeostasis regulator (Monteagudo et al., 2017). *Dot1l* polymorphism was found to be associated with hip joint cartilage thickness and OA risk in research involving 6,532 people (Castaño Betancourt et al., 2012). According to an *in vitro* study, DOT1L can methylate H3K79 of the *LEFL* and *TCF1* genes, hence reducing WNT pathway activation and causing chondrocyte hypertrophy (Monteagudo et al., 2017). *EZH2* is another histone methyltransferase discovered to modulate the WNT pathway. *EZH2*, which is elevated in OA articular chondrocytes, can increase trimethylation of the *SFRP1* promoter and is a *WNT* inhibitor, leading to hyperactivation of the *WNT/β-catenin* signaling pathway (Chen L. et al., 2016). Overexpression of *EZH2* increases the expression of *MMP13, ADAMTS5, and COLX*, *via* methylation of miR-138 promoter, resulting in cartilage breakdown (Chen L. et al., 2016). In addition to histone methylation, the demethylation process is implicated in OA as well. Jumonji domain-containing 3 (*JMJD3*), HEK27Me3 demethylase, was found at increased level in cartilage from the tibial plateau of the OA knee. Its inhibition *in vitro* by *GSK4*, significantly increases the expression of OA-related genes *MMP13* and *PTGS2*.

## Non-coding RNA

### microRNA (miRNA) and OA

Mature miRNAs bind to complementary messenger RNA (mRNA) sequences of target genes via RNA-induced silencing complexes (RISCs) or block gene expression directly. Interactions between miRNAs and highly complementary targets lead to mRNA degradation, while incomplete interactions between miRNAs and target transcripts usually lead to translation suppression. In 2008, Dimitrios et al. found that in the chondrocytes of arthroplasty patients, miR-483, miR-22, miR-377, miR-103, miR-16, miR-223, miR-2b, miR-23b, and miR509 were elevated, and miR-29a, miR-140, miR-25, miR-337, miR-210, miR-26a, and miR-373 were decreased. The genetic signatures of these 17 miRNAs clearly distinguish OA chondrocytes from normal chondrocytes, and 17 miRNA-protein pairs that may be involved in the progression of osteoarthritis were revealed by matching microRNAs and proteomics. In this study, miR-22 and miR-103 expression were favorably connected with BMI, but miR-25, and miR-337, while miR-29a expression was inversely correlated with BMI (Iliopoulos et al., 2008). Since then, numerous research has investigated the connection between miRNA expression and effector genes in OA, including inflammation, aging, transcription factors, apoptosis, autophagy, and other pathogenic events in the evolution of the illness (Zhang et al., 2015; Wang et al., 2016; D'Adamo et al., 2016; Miyaki et al., 2010). MiR-140 is a well-known miRNA associated with osteoarthritis that can be found in peripheral blood circulation or synovial fluid during the progression of OA (Miyaki et al., 2010). In articular cartilage of OA patients, the extracellular matrix is actively remodeled under inflammatory conditions, altering the local biomechanical properties of chondrocytes and accelerating the course of OA, attesting that MiR-146a has a significant function in OA inflammatory induction (Yamasaki et al., 2009). Other previously unmentioned miRNAs associated with ECM degradation include miR-137, miR-449, miR221, miR-30a, miR-19b-3p, miR-634, miR-29, miR-107, miR-497-5p, miR-26a, miR-101 (Akbari Dilmaghnai et al., 2021). Certain miRNAs, such as miR-378 in synovial fluids and let-7e in blood, might change their expression level as the disease progresses (Beyer et al., 2015; Li et al., 2016). The intra-articular injection of certain microRNAs has the ability to reverse disease progression, which will revolutionize the treatment of osteoarthritis.

### lncRNA and OA

Unlike microRNAs that rely mostly on RNA sequence complementary pairing to suppress target genes, lncRNAs operate in a significantly more sophisticated manner. lncRNAs have a unique role in a variety of gene expression regulation mechanisms, including epigenetic regulation, transcriptional regulation, and post-transcriptional regulation (Hoolwerff et al., 2020; Wang Z. et al., 2021; Statello et al., 2021).

Current research has revealed the way of axial regulation of the highly expressed lncRNA in OA tissue to the appropriate miRNA, which subsequently influences the OA target genes. HOTTIP, for instance, was engaged in the proliferation and death of OA chondrocytes via the miR-663a/FRK axis (He et al., 2021). Other lncRNA and miRNA interaction modes are more unique. Through spatial conformation, the secondary structure created by lncRNA can exert a sponge-like adsorption effect on miRNA, alter the actual contact concentration of miRNA, and the production of inflammatory genes or transcription factors in OA tissue (Chen et al., 2020a; Liu et al., 2020a; Zhang Y. et al., 2020; Zhou et al., 2021; Fan et al., 2021; Jiang et al., 2021).

## Combination of Epigenetic Factors

In the pathological process of osteoarthritis, a multitude of epigenetic variables may influence the expression of OA-related genes. For instance, during articular chondrocyte death, *DNMT3b* facilitated the downregulation of miR-29 by increasing its promoter methylation. This, in turn, led to the overexpression of *PTHLH*, a process strikingly similar to that observed in tumor disorders (Dou et al., 2020). Numerous studies have identified a connection between HDACs and miRNA cooperation patterns of OA, including *HDAC3, HDAC4, HDAC7*, and *HDAC8* (Chen et al., 2016b; Chen et al., 2016a; Mao et al., 2018; Meng et al., 2018; Zhang et al., 2019). For instance, miRNA-381 targets the 3′UTR of *HDAC4*, which in turn leads to increase in acetylation of *H3, RUNX2 and MMP13,* and ultimately result in chondrocyte hypertrophy (Chen et al., 2016a). In Table 2, a selection of the reasonably well-studied regulatory mechanisms of these models is listed.

**TABLE 2:** lncRNA which utilize with miRNA in OA progressing.

lncRNA	miRNA	Interrelated target/Regulators
SNHG14	miR-124-3-p	FSTL-1, NLRP3, TLR4/NF-κB pathway Wang B. et al. (2021)
LINC02288	miR-374a-3p	RTN3 Fu et al. (2021)
Linc-ROR	miR-138/miR-145	SOX9 Feng et al. (2021)
RNA HOTTIP	miR-663a	Fyn-related kinase He et al. (2021)
RNA RMRP	miR-206	CDK9 Lu et al. (2020)
RNA SNHG16	miR-373-3p	PPARGC1B signaling pathway (sponging miR-373-3p) Fan et al. (2021)
RNA SNHG7	miR-214-5p	PPARGC1B signaling pathway Xu et al. (2021)
RNA GAS5	miR-137	caspase-3, Bax/Bcl-2 Gao S. T. et al. (2020)
PVT1	miR-93-5p	HMGB1, TLR4, NF-κB pathway Meng et al. (2020)
RNA XIST	miR-27b-3p	ADAMTS-5 AXIS Zhu et al. (2021)
RNA NEAT1	miR-543	MMP-3, MMP-9, MMP-13, interleukin (IL)-6 and IL-8 PLA2G4A axis Xiao et al. (2021)
RNA SNHG5	miR-10a-5p	IL-1β, H3F3B axis, sponging miR-10a-5P (Jiang et al. (2021)
ARFRP1	miR-15a-5p	NF-κb, TLR4 axis Zhang G. et al. (2020)
PCAT1	miR-27b-3p	sponging miR-27b-3p (Zhou et al. (2021)
MIR4435-2HG	miR-510-3p	MMP1,MMP13, collagen II,IL17-A,<!--Soft-enter Run-on-- > p65, phosphorylated (p)-p65, IκB and p-IκB in CHON-001, sponging miR-510-3p Liu et al. (2020a)
SNHG9	miR-34a	methylation Zhang H. et al. (2020)
H19	miR-483-5p	Dusp5 Wang et al. (2020)
LOXL1-AS1	miR-423-5P	KDM5C axis, Chen K. et al. (2020)
SNHG15	miR-7	KLF4, sponging miR7a Chen et al. (2020a)
Loop LINC00511	miR-150-5P	the 3′-UTR of transcription factor (SP1) Zhang Y. et al. (2020)
XIST	miR-149-5p	DNMT3A Liu et al. (2020b)
XIST	miR-653-5p	DNMT3A Lian and Xi, (2020)
IGHCγ1	miR-6891-3p	TLR4 Zhang P. et al. (2020)
HOTAIR	miR-20b	PTEN Chen et al. (2020b)
H19	miR-106b-5p	TIMP2 Tan et al. (2020)
SNHG15	miR-141-3p	BCL2L13 Zhang X. et al. (2020)

## Conclusion

As a form of non-coding genetic information, epigenetics plays distinct regulatory roles in disease onset and progression. Epigenetics has made great strides in oncology research, and drugs targeting DNA methylation and histone deacetylases have also emerged. Numerous studies in the field of OA research have demonstrated that epigenetics regulations produce interconnected and dynamic disease progression changes. OA-related signaling pathways, transcription factors, inflammatory factors, extracellular matrix (ECM) proteins, and other variables traditionally associated with OA are all sensitive to epigenetic control to varying degrees. The combination of these genetic and epigenetic variables provides more insight into the pathophysiology of osteoarthritis. Epigenetic control can link a range of genetic and environmental factors, which could provide a holistic understanding of the etiology of osteoarthritis (OA) and guide treatment.

At present, the epigenetics of osteoarthritis focuses primarily on chondrocytes, with only two published works addressing the epigenetic manifestations of subchondral bone (Jeffries et al., 2016; Zhang et al., 2016). Zhang Y et al. discovered that not only did the subchondral bone share 111 differential methylated probes (DMPs) and 41 differential methylated genes (DMGs) with chondrocytes, but they also proposed a novel hypothesis that the subchondral compartment epigenetic changes take precedence over cartilage in the development of osteoarthritis by comparing the tibia zoning analysis (Zhang et al., 2016). Due to the differential expression of epigenetics in various tissues, subchondral bone, synovium, ligaments, and other joint tissues besides chondrocytes may contradict experimental results.

For future approaches to epigenetics in the realm of osteoarthritis, it would be prudent to investigate refined single-cell research. In cartilage tissue isolated from OA samples with variable degrees of injury and degeneration, there exist chondrocytes, macrophages, fibroblasts, and numerous other types of tissue. This heterogeneity can compromise the accuracy of current knowledge of gene function and epigenetic regulation. In the case of osteoarthritic cartilage, single-cell investigations provide more precise subtyping of diseased tissues. Embryonic cell research has demonstrated the validity of single-cell methylation investigations (Lorthongpanich et al., 2013). Using EpiTOF, histone modification investigations on a single cell are now also feasible. Adopting lanthanide metal isotopes labeled antibodies and mass spectrometry, this method allows epigenetic landscape profiling at single-cell precision (Cheung et al., 2018). In the future, these techniques could be deployed in osteoarthritis research, and this refinement will bring a fresh viewpoint to the field of osteoarthritis research. Large samples and extensive genetic and epigenetic analysis, along with prospective studies of pertinent patient histories and imaging presentations, will eventually provide solid evidence to guide the tertiary prevention and individualized and precise therapy of osteoarthritis.
